# Sonic Hedgehog Signaling in Thyroid Cancer

**DOI:** 10.3389/fendo.2017.00284

**Published:** 2017-10-30

**Authors:** Xiulong Xu, Yurong Lu, Yi Li, Richard A. Prinz

**Affiliations:** ^1^College of Veterinary Medicine, Institute of Comparative Medicine, Yangzhou University, Yangzhou, China; ^2^Department of Anatomy and Cell Biology, Rush University Medical Center, Chicago, IL, United States; ^3^Lester and Sue Smith Breast Center, Baylor College of Medicine, Houston, TX, United States; ^4^Department of Surgery, NorthShore University Health System, Evanston, IL, United States

**Keywords:** thyroid neoplasms, sonic hedgehog, cancer stem cells, phosphortidylinositol-3 kinase, MAP kinase signaling system, BRAF

## Abstract

Thyroid cancer is the most common malignancy of the endocrine system. The initiation of thyroid cancer is often triggered by a genetic mutation in the phosphortidylinositol-3 kinase (PI3K) or mitogen-activated protein kinase (MAPK) pathway, such as *RAS* and *BRAF*, or by the rearrangement of growth factor receptor tyrosine kinase genes such as *RET/PTC*. The sonic hedgehog (Shh) pathway is evolutionarily conserved and plays an important role in the embryonic development of normal tissues and organs. Gene mutations in the Shh pathway are involved in basal cell carcinomas (BCC). Activation of the Shh pathway due to overexpression of the genes encoding the components of this pathway stimulates the growth and spread of a wide range of cancer types. The Shh pathway also plays an important role in cancer stem cell (CSC) self-renewal. GDC-0449 and LDE-225, two inhibitors of this pathway, have been approved for treating BCC and are being tested as a single agent or in combination with other drugs for treating various other cancers. Here, we review the recent findings on activation of the Shh pathway in thyroid cancer and its role in maintaining thyroid CSC self-renewal. We also summarize the recent developments on crosstalk of the Shh pathway with the MAPK and PI3K oncogenic pathways, and its implications for combination therapy.

## Introduction

Thyroid cancer is the fifth most common cancer in women in the USA. Approximately 64,000 patients were newly diagnosed in 2016 (1, 2). The incidence of thyroid cancer has risen sharply in the past two decades. Much of this increase is attributed to newer and more sensitive imaging equipment and to intensive surveillance (3). Types of thyroid cancer include well differentiated papillary thyroid carcinomas (PTCs), follicular thyroid carcinomas (FTCs), medullary thyroid carcinomas (MTCs), Hürthle cell carcinomas (HTCs), and poorly differentiated or anaplastic thyroid carcinomas (ATCs) (2). PTCs account for more than 80% of all thyroid cancers (2). Surgery, thyroid hormone therapy, and radioiodine can cure most well differentiated thyroid cancers (PTC and FTC) but are much less effective treating poorly differentiated thyroid cancers. In addition, approximately 15–20% of all thyroid cancer patients will develop recurrence in their lifetime. The 10-year survival rate for patients with recurrent disease is approximately 10% (2, 4). The undifferentiated anaplastic subtype of thyroid cancer is almost always fatal, with a mean survival of only 2–6 months. There were approximately 2,000 deaths from thyroid cancer in the USA in 2016 (3).

Genetic alterations in thyroid cancer are relatively well understood (Table 1). They include *RET/PTC* rearrangements and mutations in the *RAS, RET, BRAF, PIK3CK*, and *TERT* genes (2). Mutations of these genes lead to activation of two prominent signaling pathways, the mitogen-activated protein kinase (MAPK) and phosphortidylinositol-3 kinase (PI3K) pathways. Approximately 60% of PTCs have a *BRAF* V600E mutation. *BRAF-*mutated PTCs often present with the pathological features of the classical variant or tall cell subtype. Approximately 15% of PTCs have a gene rearrangement with a frequency of *RET* > *NTRK* > others (2, 5). Those with a *RET* or *NTRK* rearrangement are mainly classical PTCs (2). Approximately 13% of PTCs have a *RAS* mutation (*NRAS* > *HRAS* > *KRAS*) and have a follicular variant feature (2). PTCs with *BRAF* mutations tend to be associated with more aggressive clinicopathologic characteristics, such as increased local invasion and distal metastasis, advanced stage at diagnosis, decreased radioiodine uptake, and increased mortality (6). The *BRAF* gene is almost never mutated in FTC (2). Instead, activation mutations of the *RAS* and *PIK3CA* (the p110 catalytic subunit of the PI3K) genes or the inactivation mutations of the *PTEN* gene frequently occur in FTC. Recent studies have shown that approximately 10% of PTCs have a *TERT* gene mutation, whereas 40% of poorly differentiated thyroid cancers and 70% of ATCs have a *TERT* mutation (7–9). ATCs are thought to progress from some well-differentiated PTCs or FTCs (2). *BRAF* and *RAS* are mutated in 45 and 24% of ATCs, respectively. The majority of ATCs harbor mutations of the *BRAF* or *RAS* gene plus the *TERT* gene (2). Understanding these genetic alterations and the activation of these signaling pathways offers unique opportunities for targeted therapy of thyroid cancer. However, due to drug resistance and crosstalk between different signaling pathways, targeted therapy often achieves only moderate or limited success. Therefore, the prevailing consensus is that combination therapies are needed to simultaneously target multiple signaling pathways to overcome drug resistance.

**Table 1:** Major genetic alterations in thyroid follicular cell carcinomas.

	*BRAF*	*RET/PTC Rearrangement*	*NKTR Rearrangement*	*RAS*	*PIK3CA*	*PPARG*	*TERT*	*ALK*	*p53*
Papillary thyroid carcinoma	60%	15%	5–13%	13%	1–3%	Rare	10%	Rare	0–5%
Follicular thyroid carcinoma	Rare	Rare	Rare	20–40%	10–15%	25–63%	17%	Rare	0–9%
Anaplastic thyroid carcinoma	45%	Rare	Rare	24%	12–23%	Rare	70%	11%	85%

## The Sonic Hedgehog (Shh) Pathway

**Figure 1:**
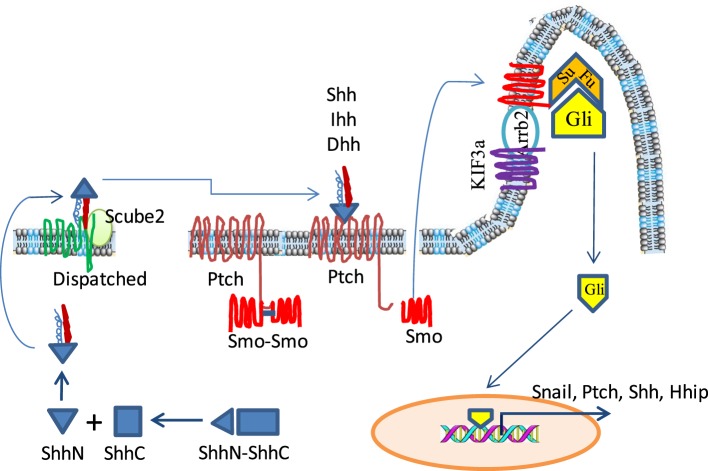
The sonic hedgehog (Shh) signaling pathway in a mammalian system. Hedgehog (HH) ligand proteins are processed in the cytosol by autoproteolytical cleavage to generate an N-terminal subunit, which is further modified by the addition of palmitoyl and cholesterol moieties. The lipidated Shh is stored in the lipid-rich microdomain on the cell surface but is released by cooperative action of Dispatched and Scube 2. In the absence of ligand binding, Patched (Ptch) restrains Smoothened (Smo) in the cytosol and keeps it as an inactive dimer. Glioma-associated oncogene (Gli) is located at the ciliary tip where it interacts with and is repressed by Suppressor of fused (SuFu). Upon HH binding, Ptch releases Smo and allows it to translocate into the cytoplasmic membrane of the ciliary tip where it cooperates with KIF3a and Arrb2 to disrupt the interaction of SuFu and Gli. Freed Gli is then translocated into the nucleus to activate the transcription of its target genes such as Snail, Shh, and Ptch.

The Shh pathway is activated by three ligands [Shh, Indian hedgehog (Ihh), and Desert hedgehog (Dhh)] that bind to their shared Patched (Ptch) receptor. These ligands are synthesized as precursor proteins, which are then cleaved to produce an N-terminal signaling protein that allows dual lipid modifications (Figure 1) (10, 11). The first modification is the addition of a cholesterol moiety on the C-terminus of cleaved hedgehog (HH), which allows HH to be retained at the plasma membrane. The second modification is mediated by HH acyltransferase, which catalyzes the addition of a palmitoyl group to the cholesterol-modified HH (11–13). Lipidated HH tends to be retained in sterol-rich membrane microdomains (14–16). Dispatched (Disp), a large multi-pass transmembrane protein, cooperates with Scube2, a secreted glycoprotein, to release the HH ligands from the plasma membrane and shield it from the aqueous microenvironment (17, 18). In addition, the lipidated HH can form monomers or multimers through cholesterol linkages (19, 20). HH-interacting protein (Hhip1) and the glycophosphatidylinositol (GPI)-linked heparin sulfate proteoglycan, Glypican-3 (Gpc3), can sequester HH, thus preventing its binding to Ptch receptor and inhibiting its activity (21–23).

Patched is a 12-pass transmembrane receptor in HH-responsive cells. In the absence of HH, Ptch constitutively represses Smoothened (Smo), a G-protein-coupled seven-pass transmembrane receptor, by preventing Smo translocation into the primary cilia (24–26). Therefore, in the absence of HH, Smo is inactive and is present in the cytoplasm as a dimer through the clustering of two amino acids in its C-terminus, arginine and asparagine (Figure 1). The Glioma-associated oncogene (Gli) proteins, including Gli1, Gli2, and Gli3, are a family of latent zinc-finger transcription factors. Gli forms complexes with the Suppressor of fused (Sufu). These complexes are located at the ciliary tip (Figure 1) (27, 28). Protein kinase A (PKA) and glycogen synthase kinase 3β (GSK3β) phosphorylate Gli2 and Gli3 but not Gli1 to create a binding site for the adaptor protein β-transducin repeat containing protein (β-TrCP) (29, 30). The Gli/β-TrCP complex is ubiquinated by the Cul1-based E3 ligase followed by partial proteasomal degradation. Truncated Gli2 and Gli3 translocate into the nucleus and usually function as transcriptional repressors through competing with Gli1 to bind the same DNA sequence (31). Since Gli1 cannot be processed in this way, it remains as a full-length transcriptional activator (32).

With HH ligand binding, Ptch1 activates the G-protein-coupled receptor kinase-2 (Grk2) to phosphorylate the adjacent domain of the C-terminus of Smo, and converts it from the inactive to an open conformation by neutralizing the electrostatic interactions of Smo dimers (33, 34) (Figure 1). HH binding to the Ptch receptor can be strengthened by several coreceptors, including CAM-Related/Downregulated by Oncogenes (Cdon), Brother of Cdon (Boc), and growth arrest specific 1 (Gas1), which form a multimolecular complex with Ptch (35, 36). HH binding also leads to Ptch1 internalization and degradation by lysosomes. Active Smo interacts with β-Arrestin (Arrb2) (37, 38) and the intraflagellar microtubule motor protein Kif3α, and translocates within the ciliary membrane where it facilitates the release of transcriptionally active full-length Gli proteins (GliA) from Sufu, thus avoiding proteasomal proteolytic cleavage and processing (37, 39). Gli1 then translocates to the nucleus and transcriptionally activates HH target genes (Figure 1).

## Crosstalk Between the MAPK and Shh Pathways

**Figure 2:**
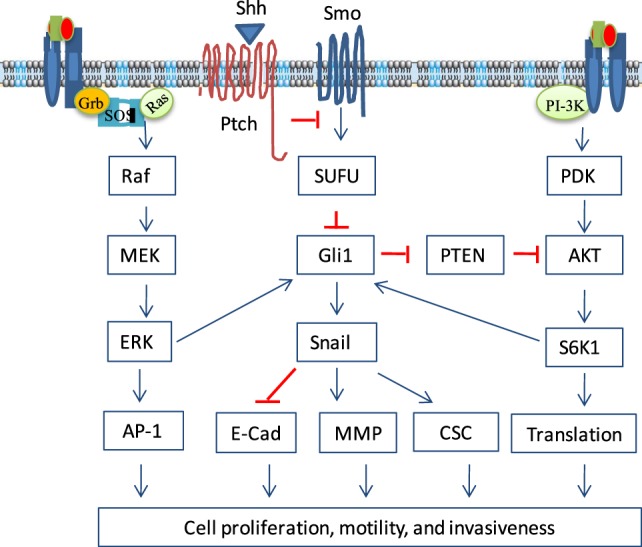
Non-canonical activation of the sonic hedgehog (Shh) pathway. Growth factor binding to their receptors activates two prominent oncogenic pathways, the phosphortidylinositol-3 kinase (PI3K) and mitogen-activated protein kinase (MAPK) pathways. In addition to the canonical activation, Gli1 can be activated by S6 kinase 1 (S6K1)-mediated phosphorylation at Serine 84, leading to nuclear translocation and induction of gene transcription. Gli1 can also be activated by ERK, probably through phosphorylation of its N-terminus by ERK2. Gli2 activity can be regulated by the MAPK pathway through increasing its stability. In addition, the MAPK pathway can activate the Shh pathway by inducing Shh expression through transcriptional upregulation. Gli1 can reciprocally activate the PI3K pathway indirectly by inducing Bmi1 expression, which represses PTEN expression. Crosstalk between the Shh and other oncogenic pathways regulates a variety of cellular functions, including cell proliferation, cell cycle progress, epithelial-to-mesenchymal transition, cell motility and invasiveness, and cancer stem cell (CSC) self-renewal.

The MAPK pathway is activated by a variety of extracellular stimuli, such as growth factors, osmotic stress, UV irradiation, reactive oxygen species, cytokines, and integrins (40) There are three parallel MAPK pathways, the classical MAPK, JNK, and p38 kinase pathways (41, 42). The cascade of these three MAPK pathways involves the activation of multiple serine/threonine kinases in the order of MAP3K → MAP2K → MAPK (40) (Figure 2). The classical MAPK pathway is activated by the binding of growth factors or cytokines to their receptor tyrosine kinases, which activate Ras through two adaptor proteins, Grb2 and SOS (43). Ras activation leads to the activation of the MAPK cascade from RAF (B-Raf, C-Raf, Raf-1) → MEK1/2 → ERK1/1 (44).

Mutations of the genes in the MAPK pathway, such as *RAS* and *BRAF*, frequently take place in a wide variety of solid and hematological malignancies (45, 46). Several studies have shown that the Gli1 transcriptional activity can be enhanced by activation of the MAPK pathway (47). For example, Riobo et al. (48) reported that the expression of Gli target genes such as Gli1 itself and Ptch is enhanced in NIH3T3 cells transfected with constitutively active MEK mutants. The N-terminal region of Gli1, though not phosphorylated by MEK or its downstream ERK kinases, is required for sensing the MAPK pathway-mediated regulation (49). A later study showed that ERK2 may be responsible for the phosphorylation of a consensus site in the N-terminus of Gli1 (50). Ji et al. (51) reported that introduction of *KRAS* V12 into an immortalized human pancreatic epithelial cell line HPDE-c7 increases Gli1 expression levels and its transcriptional activity. Whereas inhibition of the MAPK pathway by the MEK1/2 inhibitor U0126 decreases Gli1 stability and suppresses the Gli1-mediated transcriptional activity in a *KRAS*-mutated pancreatic cancer cell line. KRAS cooperates with Gli1 to induce pancreatic cancer in a mouse model (52–54). The Shh pathway is activated in pancreatic cancers in mice transgenic for *KRAS^G12D^* and *p53^R172H^* (52). Gli1 activation is required for tumor cell survival and KRAS-induced transformation in a second pancreatic mouse model (55). Inhibition of both Shh and MAPK pathways synergistically suppresses the proliferation of TE-1 gastric cancer cells (56). Inhibition of the MAPK pathway also leads to the inhibition of Gli1 transcriptional activity in an HT-29 colon cancer cell line (57, 58). Schnidar et al. (59) reported that the HH/GLI pathway cooperates with the epidermal growth factor receptor (EGFR) pathway to synergistically induce oncogenic transformation; and that pharmacologic inhibition of both EGFR and HH-Gli effectively reduces the growth of basal cell carcinoma (BCC) cell lines derived from mice with activated HH/GLI signaling. Similar to Gli1 regulation by K-Ras in pancreatic cancer, *HRAS* or *NRAS* mutation in melanoma stimulates Gli1 nuclear translocation by antagonizing the suppressive effect of SuFu through MEK1/2. Shh pathway inhibition by cyclopamine, a plant-derived teratogenic steroidal alkaloid that inhibits Smo (24–26), suppresses tumor growth in the *tyrosinase*-*NRAS^Q61K^*:*Ink4a^−/−^* mouse model of melanoma (60, 61). Moreover, melanoma cell lines with a *BRAF* gene mutation are more sensitive to sonidegib than those without a *BRAF* mutation (62). Activation of the Shh pathway is also responsible for increased expression of PDGFRα in vemurafenib-resistant melanoma cell lines *in vitro* (63). PTCs have a high frequency of *BRAF* V600E mutation (6, 64, 65). Whether simultaneous inhibition of both Shh and MAPK pathways can synergistically inhibit thyroid tumor cell proliferation and tumor growth remains to be investigated.

## Crosstalk Between the PI3K and Shh Pathways

**Figure 3:**
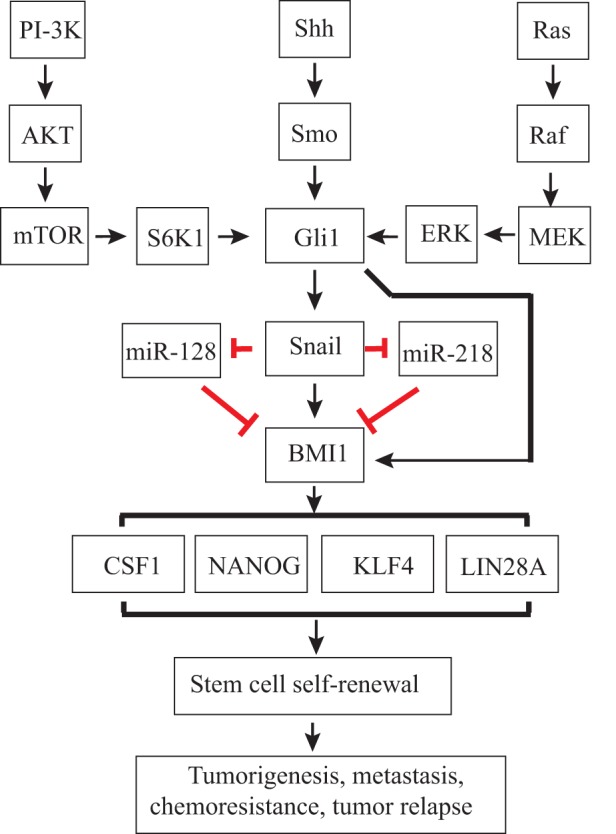
Regulation of thyroid cancer stem cell (CSC) self-renewal by the sonic hedgehog (Shh) pathway. Canonical or non-canonical Gli activation induces Snail expression. Gli1 and Snail may directly induce Bmi1 expression or indirectly induce Bmi1 expression through miRNAs, such as miR-128 or miR-218. Bmi1 is a master regulator that controls the expression of several stem cell-related genes, such as Sox2 and Nanog, and CSC self-renewal.

The PI3K pathway plays important roles in tumor initiation, growth, and metastasis (66). It is activated by growth receptor tyrosine kinases, such as the insulin receptor, EGFR, and PDGFR (67) (Figure 3). These receptor tyrosine kinases phosphorylate the p85 subunit of the PI3K. Activated PI3K catalyzes the conversion of phosphoinositol (4,5) biphosphate (PIP2) to phosphoinositol (3,4,5) triphosphate (PIP3) (68). PIP3 interacts with the Plekstrin homology domain of AKT and recruits it to the cell membrane. Membrane-bound AKT changes its conformation and opens the C-terminal kinase domain for threonine 308 (T308) phosphorylation by phosphotidylinositol-dependent kinase (PDK). mTORC2 phosphorylates AKT at serine 473 (S473), the second site in the C-terminal hydrophobic motif, and fully activates AKT. However, the PI3K-mediated AKT activation can be antagonized by PTEN (phosphatase and tensin homolog deleted on chromosome 10), which dephosphorylates PIP3 to produce PIP2 (69). AKT is inactivated by protein phosphatase 2 A (PP2A), which dephosphorylates AKT at T308 (70), and by the Plekstrin homology domain leucine-rich repeat protein phosphatases (PHLPPs) 1 and 2, which dephosphorylate AKT at S473 (71). AKT phosphorylates tuberous sclerosis protein 2 (TSC2) and alleviates its repressive effect on RheB. RheB activates the mechanistic target of rapamycin (mTOR), a serine/threonine kinase involved in the formation of two complexes, mTORC1 and mTORC2 (72). mTORC1 consists of mTOR, mLST8, Raptor, Deptor, and PRAS40 (73) and phosphorylates the elF4E-binding protein (4F-BP) and p70 S6 kinase 1 (S6K1), a serine/threonine kinase that phosphorylates the ribosomal protein S6 (73). Both 4E-BP and S6 are involved in translation initiation and protein synthesis (Figure 2). mTORC2 consists of mTOR, Rictor, mLST8, Deptor, mSIN1, and Protor, and is responsible for AKT phosphorylation at S473.

Several studies have shown that the PI3K and Shh pathways crosstalk with each other. Wang et al. (74) reported that S6K1 phosphorylates Gli1 at S84 and frees its sequestration from SuFu (Figure 2). S6K1 activation enhances Gli1 transcriptional activity and promotes its oncogenic function in esophageal cancer cell lines (74). Moreover, inhibition of both mTOR and Smo activities synergistically suppresses tumor growth (74). Upregulation of the PI3K pathway is in part responsible for drug resistance to sonidegib, a Shh pathway inhibitor used for treating medullablastoma (75). S6K1 activation induces Snail expression and epithelial-to-mesenchymal transition (EMT) in ovarian cancer cell lines (76). Gli1 activity is enhanced by AKT and by loss of tumor suppressors, such as p53 and PTEN (77). Gli1 is a key sensor that responds to both HH and an oncogenic load (77). Combined targeting of both the Shh and PI3K pathways achieves a synergistic therapeutic effect for a subgroup of chronic lymphocytic leukemia patients (78). The Shh and PI3K pathways synergistically promote the viability and growth of human PTEN-deficient glioblastomas (79). Co-inhibition of the PI3K and Shh pathways leads to mitotic catastrophy, tumor cell apoptosis, with a marked decrease of growth of PTEN-deficient glioblastomas *in vitro* and *in vivo* (79). The PI3K and Shh pathways also crosstalk in esophageal cancer (56). Cyclopamine inhibits EGF-stimulated AKT phosphorylation in TE-1 cells, an esophageal cell line; whereas Shh induces AKT phosphorylation, which is also partially inhibited by cyclopamine (56). Cyclopamine in combination with LY294002 synergistically inhibits the proliferation of three melanoma cell lines (WM-115, MeWo, and SK-Mel2) (61).

The PI3K pathway is highly activated in a variety of malignancies due to overexpression of growth factor receptor tyrosine kinases or due to mutations of the receptor tyrosine kinases (66, 80). Though PI3KCA and PTEN mutations only occur in ATCs and FTCs, the PI3K pathway is also highly activated in PTCs (68). Immunohistochemical staining and Western blot revealed AKT phosphorylation in >50% of thyroid cancers (68). Overexpression and hyperactivation of the growth factor receptor, *RAS* gene mutations, *PIK3CA* amplification, *PTEN* promoter hypermethylation, and *RET/PTC* rearrangements may all contribute to AKT activation (68). In addition, AKT nuclear localization is associated with thyroid cancer invasion and metastasis. AKT is detected in the nuclei of thyroid cancer cells, in particular in the region of tumor invasion (68).

Limited studies suggest that the PI3K and Shh pathways also crosstalk in thyroid cancer. AKT phosphorylation is decreased by inhibition of the Shh pathway with the Gli1 inhibitor GANT61 or with Shh/Gli1 knockdown, but it is increased in Gli1-overexpressing KAT-18 cells (81). Unexpectedly, cyclopamine inhibits Gli1 expression but has little effect on AKT phosphorylation in KAT-18 and SW1736 cells (81). It is possible that only Gli1 activation is responsible for AKT phosphorylation. A Smo inhibitor cannot, whereas a Gli1 inhibitor can inhibit AKT phosphorylation. Similarly, GANT61 inhibits AKT phosphorylation in embryonal and alveolar rhabdomyosarcomas (82). While these studies suggest that the Shh pathway regulates the activity of the PI3K pathway, whether the PI3K pathway also regulates the Shh pathway in thyroid cancer has not been investigated.

## Shh Signaling in Thyroid Cancer

The Shh pathway plays an important role in tumorigenesis and is a valuable molecular target for cancer therapy (24, 25, 83). Activation of the Shh signaling pathway predisposes individuals to the development of the nevoid basal cell carcinoma syndrome (NBCCS), an autosomal-dominant disorder characterized by *PTCH* mutations (84–86). *SMO* and *PTCH* mutations are found in sporadic BCC and medulloblastomas in their early stage of tumor growth (86–89). Ligand-dependent activation of the Shh pathway occurs in early stage breast, prostate, digestive tract, and small cell lung cancers (90–94). The Shh pathway promotes tumorigenesis in part by stimulating cell proliferation *via* inducing the expression of the *Cyclin* D, *N-Myc, Igf2*, and *Hes1* genes (24–26). Cyclopamine inhibits tumor cell proliferation and growth by inhibiting Smo activity (24–26).

Numerous studies suggest that the Shh pathway is involved in the growth and invasion of thyroid cancer *in vitro* and *in vivo*. We have reported that the Shh pathway is highly activated in approximately two-thirds of thyroid neoplasms and in thyroid cancer cell lines (95, 96). Shh, Ptch, Smo, and Gli1 are detected in approximately two-thirds of FTAs and PTCs and in the majority of ATC specimens (95). Greater than 77% of thyroid tumors remain simultaneously positive or negative for Shh, Ptch, Smo, and Gli1 (95). mRNAs and proteins of Shh, Ptch, Smo, and Gli1 were detected in three thyroid tumor cell lines (KAT-18, SW1736, and WRO82) (95). Inhibition of the Shh pathway by Shh or Gli1 knockdown or by Smo or Gli1 inhibitors significantly reduces cell proliferation (95). However, clinicopathological analysis shows no correlation between the activation of the Shh pathway and local invasion, distant metastasis, or tumor stage (95). By contrast, Bian et al. found that increased expression of the components of the Shh pathway is associated with increased thyroid tumor invasion and metastasis in a large number of PTC cases (97). The components of the Shh pathway are expressed in approximately 50% of anaplastic thyroid cancers and in two ATC cell lines, Hth 74 and C643 (98, 99). Both cell lines are very sensitive to cyclopamine, with the IC_50_ values between 1 and 4 µM. The components of the Shh pathway are also highly expressed in 7 MTC specimens; GDC-0449, a Smo inhibitor, inhibits the proliferation of TT cells, an MTC cell line (98, 99). These results collectively suggest that the Shh pathway is highly active in more than 50% of thyroid cancers to stimulate their cell proliferation.

## Shh Signaling in Thyroid Cancer Stem Cells (CSCs)

### Thyroid CSCs

Cancer is a very complex tissue (100). Cancer cell heterogeneity can be explained by two mutually non-exclusive stochastic and hierarchical models (100, 101). The former random model postulates that cancer development is initiated by the accumulation of genetic alterations in a single cancer cell, followed by a distinct gene mutation in a subpopulation of cells that are subsequently derived from this cell line (100, 102). The CSC model postulates that cancer comprises tumor cells in a hierarchy of cellular differentiation. The ability of CSCs to renew themselves confers CSC ability to resist chemotherapy, to metastasize, and to develop recurrent disease (103, 104). Thus, even if the initial response to chemotherapy or radiation is encouragingly robust, the patient may not be cured as long as CSC survives. Thus, these features of CSCs could explain well-observed but poorly understood phenomenon in cancer biology, including metastasis, recurrence, and therapeutic resistance (100).

Cancer stem cells are characterized by their remarkable capacity to develop tumors when implanted in immunodeficient mice and by their capacity to grow sphere-like aggregates in ultra-low attachment plates in the serum-free medium (100). Several markers have used to identify a unique group of stem cells in normal thyroid follicles and CSCs in thyroid cancers (101, 105–108). Stem cells identified in normal thyroid tissue in mice and humans are a side population in flow cytometry with Hoechst 33342 staining. These cells exhibit stem/progenitor cell-like characteristics, including the expression of stem cell markers, such as nucleostemin and Oct4, but do not express cell differentiation markers, such as thyroid peroxidase, thyroglobulin, and thyroid stimulating hormone receptor (109, 110). This side population identified in thyroid cancer cell lines has great capacity to form thyrospheres. It also expresses ATP-binding cassette sub-family G member 2 and exhibits increased clonality and invasive potential as well as drug resistance (111, 112). CD133 marks CSCs in several other tissues, but whether it could be used as a marker for thyroid CSCs remains controversial (101).

Aldehyde dehydrogenase (ALDH) is a reliable marker of CSCs in several types of malignancies. ALDH-positive cells isolated by flow cytometry can be analyzed for their tumor-initiating capacity. Todaro et al. reported that thyroid CSCs constitute a unique population (1–3%) with highly invasive and metastatic behavior in different types of thyroid cancers (113). Poorly differentiated or undifferentiated thyroid cancers contain a higher percentage of ALDH-positive CSCs than benign adenomas and well differentiated thyroid cancers (113). ALDH^+^ CSCs can expand indefinitely *in vitro* as tumor spheres and retain their tumorigenic potential when implanted in immunocompromised mice. Several hundred CSCs injected into the thyroid of immunocompromised mice develop into thyroid cancer that recapitulate the behavior of the parental tumor, including the aggressive metastatic features of undifferentiated thyroid carcinomas (113). Consistent with these observations, Hardin et al. (114) recently characterized two ALDH^high^ clones derived from an ATC cell line, THJ-16T, and found that these clones have a much higher capacity to form thyrospheres than their parental cells and are highly tumorigenic in immnocompromised mice. By contrast, Shimamura et al. (115) showed that inhibition of ALDH activity with a specific inhibitor or by siRNA knockdown reduces ALDH-positive cells but does not significantly lower the number and growth of thyrospheres of four thyroid cancer cell lines (FRO, ACT1, KTC3, and 8505C), suggesting that ALDH may be just a marker for thyroid CSCs and may not have a functional role in thyroid CSC self-renewal. Ma et al. (116) showed that SSEA-1 is also a specific marker for thyroid CSCs; and that SSEA-1-postive thyroid CSCs express high levels of stem cell-related genes, such as Nanog, Sox2, and Oct4, and are resistant to 5-fluorouracil cytotoxicity. These authors also showed that an injection of 10,000 SSEA-1-positive T238 cells, an ATC cell line, was needed to develop cancer into athymic mice (116).

### Regulation of Thyroid CSC Self-Renewal by the Shh Pathway

The Shh pathway has been implicated in regulating the self-renewal of CSCs (117). In breast cancer, the Shh pathway plays a critical role in maintaining mammary stem cells (118, 119). The mRNA levels of the genes in the Shh pathway, including *PTCH1, GLI1*, and *GLI2*, are elevated in CD44^+^/CD24*^−^*^/low^Lin*^−^* breast CSCs (119). The Shh pathway is also required for the maintenance of self-renewal of embryonal rhabdomyosarcoma CSCs (120). Inhibition of the Shh pathway by GANT61 or by siRNA suppresses the formation of tumor spheres *in vitro* and the development of embryonal rhabdomyosarcoma *in vivo* (120). Activation of the Shh pathway in multiple myeloma cell lines NCI-H929 and KMS12 stimulates the self-renewal and expansion of CD138^+^CD19*^−^* CSCs; whereas inhibition of the Shh pathway by cyclopamine or by the Shh neutralizing antibody 5E1 decreases the clonal capacity of multiple myeloma cell lines and CD138*^−^* cells through the induction of plasma differentiation (121). Loss of Shh signaling by genetically disrupting *SMO* results in the inhibition of BCR-ABL expressing leukemic stem cells and prolongs their survival (122, 123). The Shh signaling pathway is also highly activated in glioblastoma CSCs, whereas cyclopamine or siRNA directed against the pathway components results in the loss of tumorigenic potential (124, 125). Thus, the Shh pathway dictates the fate of CSCs, including self-renewal and differentiation (117).

Studies by our group suggest that the Shh pathway is involved in regulating thyroid CSC self-renewal (126). Suppression of the Shh pathway by Shh or Gli1 knockdown in KAT-18 thyroid cancer cell line leads to decreased size and number of thyrospheres, whereas Gli1 overexpression leads to increased number and size of thyrospheres. Malaguarnera et al. (127) reported that insulin receptor and insulin-like growth factor receptor activation stimulates the formation of thyrospheres of normal and thyroid cancer cells and induces the expression of stemness-related genes. Chen et al. (128) reported that metformin inhibits the proliferation of thyroid carcinoma cells, suppresses the self-renewal of CSCs, and potentiates the therapeutic effect of chemotherapeutic agents. Mechanistic studies suggest that activation of AMPK by metformin and subsequent inhibition of mTOR is responsible for this inhibitory effect on cell proliferation and thyroid CSC self-renewal (128). It is highly likely that the stimulatory effect of insulin and the inhibitory effect of metformin on thyroid CSC self-renewal may hinge on the activation of the Shh pathway through its crosstalk with the PI3K and MAPK pathways.

A hallmark of CSCs is their ability to resist chemo- and radiation therapy (129). Overexpression of Gli1 in KAT-18 cells significantly increases the number of surviving colonies after irradiation, compared to vector-transfected control cells. CSCs are highly invasive and metastatic due to the expression of several molecules involved in tumor cell motility and invasion. For example, CSCs express high levels of CXCR4, a chemokine receptor for CXCL12/SDF1 ligand which facilitates bone metastasis (130). CSCs that survive chemo- and radiation therapy play a critical role in tumor recurrence and metastasis (129). Todaro et al. showed that c-Met and AKT are highly activated and required for stimulating thyroid CSC invasion and metastasis (113). Williamson et al. (81) showed that suppression of the Shh pathway by miRNA targeting either Shh or Gli1 in a KAT-18 anaplastic cancer cell line decreases motility and invasiveness in Matrigel. By contrast, Gli1 overexpression in KAT-18 cells increases motility and invasive potential, compared to the cells transfected with the empty expression vector (81). These observations suggest that activation of the Shh pathway stimulates the motility and invasiveness of thyroid CSCs.

### Mechanisms by Which the Shh Pathway Regulates CSC Self-Renewal

The mechanisms by which the Shh pathway maintains the self-renewal of stem cells are not fully understood. Numerous studies suggest that the Shh pathway promotes CSC self-renewal by inducing the expression of the stemness-related genes. Activation of the Shh pathway promotes breast CSC self-renewal by inducing the expression of the polycomb gene Bmi1, a master regulator of CSCs (131). Cyclopamine reduces the capacity of neurosphere formation and decreases the expression of Nanog, Sox2, and Oct4 in glioblastoma cell lines (132). Inactivation of the Shh pathway by Huaier extract reduces the number of CD44^+^/CD24*^−^* cells and decreases the levels of stem cell markers (OCT4, NESTIN, and NANOG) (133). Inhibition of the Shh pathway by cyclopamine leads to the downregulation of NANOG mRNA in a HCT-116 colon cancer cell line and decreases tumor spheres (134). Epigallocatechin-3-gallate (EGCG), an active compound in green tea that inhibits the expression of the components of the Shh pathway (Smo, Ptch, Gli1, and Gli2) and Gli transcriptional activity, inhibits the self-renewal capacity of pancreatic CSCs by inhibiting the expression of pluripotency maintaining transcription factors (Nanog, c-Myc, and Oct4) (135). Mechanistic studies suggest that Sox2 is transcriptionally upregulated by Gli1 binding to a cis-element in the SOX2 promoter (136).

Snail is a transcriptional factor whose expression is induced by Gli1 (137, 138). Snail is best known for its role in inducing EMT (139), but emerging evidence suggests that Snail plays an important role in regulating CSC self-renewal (139). Snail expression in immortalized mammary epithelial cells leads to the enrichment of CD44^hi^CD24^lo^ breast CSCs (140). Snail expression in a human squamous cell carcinoma cell line induces EMT and CSC-like properties (141). Increased Snail expression in ComBit transgenic mice leads to the spontaneous development of thyroid cancer and increases the thyroid cancer incidence rate after irradiation (142). Heiden et al. (126) reported that activation of the Shh pathway leads to increased Snail expression in thyroid cancer cell lines, and that suppression of Snail expression by siRNA decreases the number of ALDH^High^ thyroid CSCs in SW1736 and KAT-18 cells, two anaplastic thyroid cancer cell lines. These findings suggest that Snail plays a critical role in the Shh pathway-mediated maintenance of CSC self-renewal in ATC cell lines. Consistently, Ma et al. (116) reported that Snail expression is significantly higher in SSEA-1-positive thyroid CSCs than in SSEA-1-negative non-CSCs. Intriguingly, Yasui et al. (143) reported that Snail overexpression in an ACT-I thyroid tumor cell line increases the number of thyrospheres but decreases the number of ALDH^High^ cells. Baquero et al. (144) reported that BRAF V600E mutation leads to increased Snail expression and decreased E-cadherin expression in thyroid cancer cell lines. Though the underlying molecular mechanisms are not clear, it is highly likely that non-canonical Gli1 activation through the MAPK pathway may be responsible for BRAF mutation-induced Snail expression (Figure 3).

Bmi1 is a member of the polycomb gene group that regulates gene expression by chromatin modification (145). Bmi1 inhibits PTEN expression in nasopharyngeal cancers, subsequently activating AKT (146). Bmi1 stimulates the proliferation of hepatocellular carcinomas by suppressing INK4A/ARF gene expression (147). Bmi1 stimulates CSC self-renewal in hepatocellular carcinomas (147, 148), pancreatic cancer (149, 150), and head and neck squamous cell carcinomas (151, 152). Artemisinin, an antimalarial drug, inhibits tumor cell proliferation of head and neck squamous cell carcinomas by inhibiting Bmi1 expression (153). A recent study showed that Bmi1 induces an invasive signature that promotes metastasis and chemoresistance in melanoma (154).

Bmi1 is an important target gene of the Shh pathway. Activation of the Shh pathway induces Bmi1 expression in medulloblastoma and breast cancer (131, 155). Gli2 overexpression in mammosphere-initiating cells results in the production of ductal hyperplasia (131). Modulation of Bmi1 expression in mammosphere-initiating cells alters mammary development in a humanized non-obese diabetic-severe combined immunodeficient mouse model (131). Using chromatin immunoprecipitation assay, Wang et al. reported that Gli1 directly binds the promoter of Bmi1 in medulloblastoma (155). Gopinath et al. (156) reported that cathespin and uPAR proteinase induce Sox2 and Bmi1 expression and promotes the self-renewal of glioma CSCs by Gli1. Liu et al. (131) showed that activation of the Shh pathway induces Bmi1 expression in breast cancer through Gli1. Taken together, these observations suggest that the Shh pathway regulates the expression of Bmi1 by Gli1 directly or indirectly by multiple pathways (Figure 3).

Anaplastic thyroid carcinomas contain a higher percentage of CSCs than other types of thyroid cancer (113). Several CSC-related genes, *SOX2, SOX4, NANOG, c-MYC*, and *ABCG*, are highly expressed in ATC (157). Some of these molecules, in particular, Sox2, Nanog, CD133, and ABC G2, are expressed in much higher levels in thyrospheres from fresh thyroid tumors than in monolayers (127) and are expressed at higher levels in ALDH^+^ than ALDH*^−^* thyroid cancer cell lines (114). Ferretti et al. (154) recently reported that Bmi1 confers resistance to a B-Raf inhibitor by activation of the non-canonical Wnt pathway in melanoma and that a Bmi1-induced gene signature predicts metastasis and the clinical outcome of melanoma patients. Our ongoing studies suggest that Bmi1, Sox2, and Nanog are highly expressed in thyroid cancer and can be regulated by the Shh pathway. Thus, the Shh pathway may regulate thyroid CSC self-renewal by inducing the expression of stem cell-related genes through Bmi1.

## Implications and Perspectives

The Shh pathway is activated due to gene mutations or overexpression of the components of the Shh pathway, or due to cross-activation by other signaling pathways in a wide range of malignancy (33, 158). The Shh pathway is also important in the maintenance of CSC self-renewal (16). Since CSCs play essential roles in tumor initiation, drug resistance, recurrence, and metastasis, the inhibitors of the Shh pathway have been explored for treating various cancers. GDC-0449 (Vismodegib/Erivedge) and LDE-225 (Erismodegib/Sonidegib/Odomzo), two Smo inhibitors, were approved in 2012 and 2015, respectively, by the Federal Drug Administration for treating metastatic or locally invasive BCC (159). These two drugs, along with a dozen other inhibitors targeting the Shh pathway, are in various stages of clinical trials as mono- or combination therapy for a variety of malignancies (117, 158, 159). These clinical trials are promising but have achieved only modest responses (159). Better understanding of the underlying cell biology is needed to improve the efficacy of the inhibitors of the Shh pathway. In particular, most inhibitors in the Shh pathway target Smo. Non-canonical Gli1 activation by the PI3K and MAPK pathways may bypass Smo inhibition and may be responsible for the poor performance of Smo inhibitors in clinical trials (160, 161).

Although several studies suggest that the Shh pathway is highly activated in thyroid neoplasms and plays an important role in thyroid tumor cell proliferation and CSC self-renewal, many critical questions remain unanswered. First, the role of the Shh pathway in thyroid CSC self-renewal and tumor initiation has not been investigated *in vivo*. It remains unclear if blockade of the Shh pathway will eliminate CSCs and prevent thyroid tumor metastasis. Second, whether the Shh pathway is involved in thyroid tumor initiation or facilitates tumor development initiated by oncogenes, such as mutant *BRAF, RAS*, or *RET*, needs to be verified in a transgenic mouse model. Third, the crosstalk between the Shh and PI3K/MAPK pathways needs to be more thoroughly investigated. Better understanding of the interaction between the Shh and other oncogenic pathways will help design novel combination therapies for poorly differentiated or anaplastic thyroid cancers. Fourth, exactly how the Shh pathway regulates the self-renewal of CSCs in the thyroid and other organs remains vague and needs to be studied in detail. Finally, Smo and Gli1 inhibitors have not been tested in clinically relevant models such as patient-derived orthotopic xenografts. Better understanding of how the Shh signaling pathway regulates CSC self-renewal may offer unique opportunities for thyroid cancer therapy.

## Author Contributions

XX conducted literature review and wrote the manuscript. YLu contributed to the conception and design of the work in the manuscript. YLi contributed to review, discussion, and conception of the work. RP critically read, revised, and edited the manuscript.

## Conflict of Interest Statement

The authors declare that the research was conducted in the absence of any commercial or financial relationships that could be construed as a potential conflict of interest.
